# Educational interventions to improve detection and management of cognitive decline in primary care—An Italian multicenter pragmatic study

**DOI:** 10.3389/fpsyt.2022.1050583

**Published:** 2022-11-24

**Authors:** Gemma Lombardi, Elena Chipi, Domenico Arenella, Ambra Fiorani, Giovanni Battista Frisoni, Simona Linarello, Chiara Montanucci, Cristina Muscio, Irene Pacifico, Silvia Pelizzari, Daniela Perani, Fabrizio Piras, Luca Rozzini, Sandro Sorbi, Gianfranco Spalletta, Fabrizio Tagliavini, Pietro Tiraboschi, Lucilla Parnetti, Graziella Filippini

**Affiliations:** ^1^IRCCS Fondazione Don Carlo Gnocchi, Florence, Italy; ^2^Centre for Memory Disturbances, Section of Neurology, Lab of Clinical Neurochemistry, Department of Medicine and Surgery, University of Perugia, Perugia, Italy; ^3^Carlo Besta Foundation and Neurological Institute, Milan, Italy; ^4^Laboratory of Neurology, IRCCS Istituto delle Scienze Neurologiche di Bologna, Bologna, Italy; ^5^Laboratory of Epidemiology and Neuroimaging, IRCCS San Giovanni di Dio - Fatebenefratelli, Brescia, Italy; ^6^Memory Clinic, Geneva University Hospitals, Geneva, Switzerland; ^7^Dipartimento dell’Integrazione, AUSL Bologna, Bologna, Italy; ^8^ASST Bergamo Ovest - Azienda Socio Sanitaria Territoriale di Bergamo Ovest, Bergamo, Italy; ^9^Laboratory of Neuropsychiatry, IRCCS Santa Lucia Foundation, Rome, Italy; ^10^Centro per i Disturbi Cognitivi e le Demenze, Spedali Civili di Brescia, Brescia, Italy; ^11^Division of Neuroscience, San Raffaele Scientific Institute, San Raffaele University, Milan, Italy; ^12^Section of Psychology - Department of Neuroscience, Psychology, Drug Research and Child’s Health (NEUROFARBA), University of Florence, Florence, Italy; ^13^Division of Neuropsychiatry, Department of Psychiatry and Behavioral Sciences, Baylor College of Medicine, Houston, TX, United States

**Keywords:** cognitive decline, dementia, primary care, educational intervention, care pathway, family physician

## Abstract

**Introduction:**

Timely detection of cognitive decline in primary care is essential to promote an appropriate care pathway and enhance the benefits of interventions. We present the results of a study aimed to evaluate the effectiveness of an educational intervention addressed to Italian family physicians (FPs) to improve timely detection and management of cognitive decline.

**Materials and methods:**

We conducted a pre-post study in six Italian health authorities (HAs) involving 254 FPs and 3,736 patients. We measured process and outcome indicators before the intervention (1 January 2014 to 31 December 2016) and after the intervention (1 January 2018 to 31 December 2019). One interactive face-to-face session workshop was delivered by local cognitive disorders and dementia specialists and FP advisors at each HA, in the period September 2017–December 2017. The session focused on key messages of the local Diagnostic and Therapeutic Care Pathway (DTCP) or regional guidelines: (a) the role of the FP for a timely suspicion of cognitive decline is fundamental; (b) when cognitive decline is suspected, the role of the FP is active in the diagnostic work-up; (c) FP’s knowledge on pharmacological and non-pharmacological interventions is essential to improve the management of patients with cognitive decline.

**Results:**

An overall improvement in diagnostic procedures and management of patients with cognitive decline by FPs after the intervention was observed. The number of visits per year performed by FPs increased, and the time interval between the first FP consultation and the diagnosis was optimized. Neuroleptic use significantly decreased, whereas the use of benzodiazepines remained steadily high. Non-pharmacological interventions, or use of support services, were underrepresented even in the post-intervention. Differences among the participating HAs were identified and discussed.

**Discussion:**

Results from this study suggest the success of the educational intervention addressed to FPs in improving early detection and management of cognitive decline, highlighting the importance to continue medical education in this field. At the same time, further initiatives of care pathway dissemination and implementation should promote strategies to enhance interactions between primary and secondary care optimizing the collaboration between FPs and specialists.

## Introduction

Dementia is a chronic, progressive syndrome affecting cognitive and functional abilities, representing one of the major causes of disability and dependency among older people.

Based on the high global prevalence and the economic impact for societies, the World Health Organization recognized dementia as a public health priority (1). In Italy, the estimated number of people with dementia is more than one million and more than three million Italians are directly or indirectly involved in the assistance of patients (2).

Timely suspicion of dementia and referral to specialized healthcare services are essential proceedings to promote an appropriate care pathway in patients with cognitive decline, take charge of persons with dementia and enhance the benefits of interventions. Since family physicians (FPs) act as gatekeepers of the care pathway, they should have the capacity to detect early cognitive decline, provide information and appropriate referral to specialists, and reduce the use of harmful or ineffective interventions (3). Despite the attention to a timely diagnosis, in Italy and other European countries dementia is under-recognized and under-managed in primary care settings (4–6), and cognitive decline is often not mentioned in the medical records until it is indicative of a serious condition (7). A previous experience from a study (the REMIND study) in the Health Authority (HA) of Milan in Northern Italy revealed difficulties in the ability of FPs to early recognize cognitive decline and provide appropriate referral to a specialized service. Moreover, patients and their carers complained of poor communication at the time of referral (4). Similar data emerged from a survey conducted on FPs in Southern Italy, where several problems in properly recognizing early symptoms of cognitive decline and referring timely to specialists were documented (8).

FPs also have a key responsibility to manage multimorbidity and polypharmacotherapy avoiding ineffective or harmful treatments. In this regard, even if the use of neuroleptics and benzodiazepines has been associated with a worse outcome in dementia (9, 10), these medicines are still largely prescribed in Italy (11, 12).

In October 2014, the Italian Ministry of Health formulated the first “Dementia National Plan” providing directive indications for promoting and improving interventions regarding dementia, including “shared activities involving family physicians and carers” (13–15). In response to these indications, we conducted a before-after study in order to investigate the feasibility and effectiveness of an educational intervention addressed to FPs to promote their awareness of dementia, improve timely detection of cognitive decline, patient referral to specialists, and appropriateness of interventions.

The study was conducted in six HAs of five Italian regions and was part of the NET-2011-02346784-5 project funded by the Italian Ministry of Health.

## Materials and methods

### Study design

**FIGURE 1:**
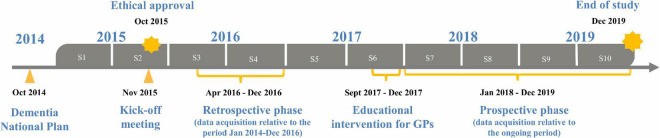
NET-2011-02346784-WP5 study design, from 2015 to 2019, each year of study consisting of two semesters (S).

The study was conducted in the HAs of Milan and Brescia (Lombardy), Bologna (Emilia-Romagna), Florence (Tuscany), Rome (Lazio), and Perugia (Umbria) involving qualified research and university centers (Supplementary Table 1), and included three sequential phases: a retrospective one between April 2016 and December 2016, an educational intervention spanning a 4-month period (September 2017–December 2017), and a prospective phase between January 2018 and December 2019 (Figure 1). We selected HAs in different Italian regions, in order to capture variation in health and social-health services for people with dementia.

The study was reviewed and approved by the ethical committee of Carlo Besta Foundation and Neurological Institute in Milan and the ethical committees of all participating HAs. The participants provided their written informed consent to be included in the study. All procedures performed in the study were in accordance with the Helsinki declaration.

### Participating family physicians

In each HA, a local research team comprising a study coordinator, specialists of the Centers for Cognitive Disorders and Dementia (CCDDs), FP advisors and HA representatives was established. A kick-off meeting was held in November 2015, at the Carlo Besta Foundation and Neurological Institute to share objectives, working plan, and evaluate available resources including local Diagnostic and Therapeutic Care Pathway (DTCP) or regional guidelines for dementia (Supplementary Table 1). Recruitment of FPs was conducted using purposive sampling to make the most out of limited resources in each participating HA. Volunteers FPs were contacted for inclusion by the study coordinator at each HA and selected based on their interest in improvement in the diagnosis and management of dementia care, their availability to attend the educational intervention and collect retrospective and prospective data. Forty volunteer FPs in each HA (30 in Perugia) participated. Participating FPs reported they took care of about six new patients with cognitive decline each year. Based on this number, we expected to include a minimum of 1,380 patients in both retrospective and prospective phases.

### Participants

Eligibility criteria included adults without limit of age who consulted their FP because aware and concerned about the onset of cognitive or behavioral and psychological symptoms. Participants were recruited also in cases of symptoms referred by a family member, someone who knew the person well or first suspected by the FP. Patients were ineligible if they had participated in any clinical trial within 3 months of recruitment, were residents outside the local HA, or had high comorbidities requiring a priority dedicated care pathway, which could impact the DTCP for dementia. The comorbidity was assessed using the Cumulative Illness Rating Scale (CIRS), an instrument providing independent information on 14 areas corresponding to body systems. Rating is made on a 5-point “degree of severity” scale, ranging from “none” (=1) to “extremely severe” (=5) for each area. We defined “high comorbidity” as a score of 4 or 5 in at least one of the 13 areas of CIRS, excluding the psychiatric/behavioral area (16).

### Data collection

FPs identified eligible patients through a 36-month period before the educational intervention, from 1 January 2014 to 31 December 2016, and a 24-month period after the intervention, from 1 January 2018 to 31 December 2019. Any overlap between patients recruited in the pre- and post- intervention period was avoided by FPs and strictly checked by the study personnel. FPs extracted data from medical records using two predefined data collection forms in an Excel spreadsheet, one form on the patient’s first visit for cognitive or behavioral problems and one on follow-up. They collected detailed information on comorbidities, diagnostic procedures including cognitive and mood examination tests as well as blood tests and neuroimaging, referral to a CCDD specialist, number of annual visits for cognitive decline performed by the FP, diagnosis during follow-up and the timing for diagnosis, prescription of medicines for cognitive or behavioral symptoms, and non-pharmacological interventions such as day center participation, cognitive or physical rehabilitation. The study coordinator visited the FPs regularly to oversee data collection and quality. All the anonymized electronic data were sent to the Carlo Besta Foundation and Neurological Institute in Milan where they were validated and combined into a central database. Data access was protected by user name and password to which only the study coordinator and the data manager had access.

### Educational intervention

One interactive face-to-face session workshop was delivered by local CCDD specialists and FP advisors at each HA. The content was shared and agreed among participating HAs. The session focused on the local DTCP’s key messages, namely:

a) FPs are typically the first contact for patients and they are responsible for their initial assessment, including timely recognition of cognitive decline or behavioral symptoms, appropriate referral to specialists, and ensuring access to health and social-health services;

b) FPs have an active role in the diagnostic work-up by using simple cognitive and mood examination tests and prescribing blood tests and brain MRI or CT scan. These exams are needed to rule out reversible causes of cognitive decline and improve referral to CCDD specialists when a neurodegenerative origin is the main diagnostic hypothesis;

c) FPs should avoid the prescription of neuroleptics and benzodiazepines which are potentially harmful treatments for patients with dementia; alternative non-pharmacological strategies should be considered and proposed.

In Italy, FPs are not allowed to start prescribing drug treatment for dementia, thus no details on their use were provided in the course, except on their possible side effects.

The workshop involved a combination of didactic lectures and small group discussion, including practice exercises on the use of cognitive and mood examination tests: the Mini-Mental-State-Examination (17) in Milan, Brescia, Bologna and Rome; the Mini-Cognitive test (18) in Florence, and the Basic Italian Cognitive Questionnaire (19) in Perugia; the 15 items Geriatric Depression Scale (20) in all HAs. Participating FPs received printed resources on the instructions provided throughout the workshop. CCDD specialists were available upon FPs’ request to discuss the management of patients with cognitive decline or behavioral symptoms throughout the study.

### Study outcomes

Outcomes were based on indicators recommended in the “Guidance on Integrated Care Pathway for People with Dementia” released by the Italian Ministry of Health to support health authorities in the development of DT dedicated to people with dementia (21). These indicators represent one of the tools for evaluating the applicability of a DTCP and the deviation between the reference DTCP and the one locally implemented.

Process indicators were the proportion of recruited patients who received:

• Two or more visits per year by FP for cognitive assessment (FP assessed the presence of cognitive/behavioral changes and their potential impact on daily life basing the information on the patient’s history, collecting information directly from the patient, a family member, or from someone who knew the person well. The visits also included a physical examination, and eventually investigations to identify comorbid conditions and to exclude reversible causes of cognitive and functional impairment).
• At least one cognitive examination test (Mini-Mental-State-Examination, Mini-Cognitive test, or Basic Italian Cognitive Questionnaire, according to local DTCP).
• At least one mood examination test (15 item Geriatric Depression Scale).
• Blood tests (complete blood count, glucose, TSH, electrolytes, creatinine, GOT, GPT, folate, and vitamin B12).
• Brain CT or MRI.
• At least one CCDD specialist consultation (neurologist, geriatrician, psychiatrist).

Outcome indicators included the time interval between the first suspicion of cognitive decline by FP and diagnosis and the proportion of patients who received:

• a baseline diagnosis of cognitive decline that was not confirmed during follow-up
• A timely detection of cognitive decline that was confirmed during follow-up
• Prescriptions of neuroleptics and benzodiazepines
• Participation in cognitive/physical rehabilitation or day center, on FP advice.

### Analysis

All data entered into Microsoft Office Excel (2012) spreadsheets. Analyses were undertaken in Excel and Stata (Version 12). Simple summary statistics were used to assess observed outcome measures and to compare pre-intervention to post-intervention outcomes. We calculated the percentage, mean (SD, IQR) and their respective 95% CIs. Comparison among outcomes between pre- and post-intervention was performed using the χ2 test for categorical variables and unpaired *t*-test for continuous variables, and the level of significance was set at a *p*-value <0.05. Analyses were carried out for all HAs combined and for each HA separately.

## Results

254 voluntary FPs from the six HAs entered and completed the study. They yielded valid data from 3,736 patients, 1,708 pre-intervention and 2,028 post-intervention. A higher proportion of FPs was male (65%), older than 55 years of age (69%), which are similar proportions compared with the national workforce of FPs (22, 23). Specialists in family medicine were over-represented in Brescia (41%) compared with FPs in the other HAs (range from 12 to 18%).

Table 1 shows the baseline characteristics of patients identified by FPs as having cognitive decline, stratified by pre- and post-intervention: mean age 79 years (SD 8), 37.6% men and 62.4% women, mean CIRS total score 6 (SD 4). Profiles of age, gender and CIRS scores were similar in the pre- and post-intervention. The mean length of follow-up was shorter in post-intervention (11 months, IQR 9–13) compared with pre-intervention (18 months, IQR 11–25). Baseline characteristics of patients among HAs were reported in Supplementary Table 2. Compared with the other HAs, patients in Rome were younger and those in Milan and Rome had lower CIRS scores. Process indicators are shown in Table 2. The proportion of patients receiving two or more visits per year by their FP for cognitive assessment increased from 33.3% (95% CI 31.1–35.6%) pre-intervention to 54.5% (95% CI 52.3–56.7%) post-intervention (*p* < 0.001), and the proportion of patients examined with a cognitive test increased from 6.5% (95% CI 5.4–7.8%) pre-intervention to 87.9% (95% CI 86.4–89.3%) post-intervention (*p* < 0.001). FPs did not use any test to examine mood before the educational intervention, whereas 60.3% (95% CI 58.1–62.4%) of patients were examined with the 15 items Geriatric Depression Scale post-intervention. Prescription of blood tests increased from 79.5% (95% CI 77.5–81.4%) to 84.9% (95% CI 83.3–86.4%) (*p* < 0.001), and prescription of a brain CT or MRI increased from 32.3% (95% CI 30.0–34.5%) to 48.1% (95% CI 45.9–50.3%) (*p* < 0.001). The proportion of patients referred to a CCDD specialist decreased from 89.8% (95% CI 88.3–91.2%) pre-intervention to 81.3% (95% CI 79.6–83.0%) post-intervention (*p* < 0.001).

**TABLE 1:** Baseline characteristics and follow-up of patients identified by FPs as having cognitive decline in six health authorities of five Italian regions, total and stratified by pre- and post-educational intervention.

	Total N. 3,736	Pre-intervention N. 1,708	Post-intervention N. 2,028	*P*-value*
**Gender, No. (%)^†^**				
Men	1,406 (37.6)	615 (36.0)	791 (39.0)	0.2
Females	2,330 (62.4)	1,093 (64.0)	1,237 (61.0)	0.1
Age, mean (SD), years	78.9 (8.0)	78.9 (8.0)	79.0 (7.0)	1.0
IQR	73.6–84.4	73.6–84.4	74.3–83.7	
CIRS total score, mean (SD)	6.0 (4.0)	6.0 (4.0)	6.0 (4.0)	1.0
IQR	3.3–8.7	3.3–8.7	3.3–8.7	
Follow-up (months), mean (SD)	–	18.0 (10.2)	11.0 (2.7)	<0.001
IQR		11.1–24.9	9.2–12.8	
**Healthcare region No. (%)^†^**				
Milan, Lombardy	1,263 (33.8)	407 (23.8	856 (42.2)	–
Brescia, Lombardy	549 (14.7)	323 (18.9)	226 (11.1)	
Bologna, Emilia-Romagna	556 (14.9)	294 (17.2)	262 (12.9)	
Florence, Tuscany	546 (14.6)	255 (14.9)	291 (14.3)	
Rome, Lazio	480 (12.8)	240 (14.1)	240 (11.8)	
Perugia, Umbria	342 (9.2)	189 (11.1)	153 (7.5)	

FPs, family physicians; SD, standard deviation; IQR, interquartile range; CIRS, Cumulative Illness Rating Scale. ^†^Percentages shown are by column. *p-value referred to comparison between pre- and post-intervention.

**TABLE 2:** Number (%, 95% CI) of patients prescribed diagnostic tests in primary care and referral to a CCDD specialist for cognitive decline, in the pre- and post-intervention.

	Pre-intervention N. 1,708	Post-intervention N. 2,028	*P*-value*
≥2 visits per year for cognitive assessment	569 (33.3, 31.1–35.6)	1,106 (54.5, 52.3–56.7)	<0.001
At least one cognitive test	111 (6.5, 5.4–7.8)	1,783 (87.9, 86.4–89.3)	<0.001
At least one GDS 15^†^	–	1,222 (60.3, 58.1–62.4)	–
Blood tests	1,358 (79.5, 77.5–81.4)	1,722 (84.9, 83.3–86.4)	<0.001
Brain CT or MRI	551 (32.3, 30.0–34.5)	976 (48.1, 45.9–50.3)	<0.001
Referral to a CCDD specialist	1,534 (89.8, 88.3–91.2)	1,649 (81.3, 79.6–83.0)	<0.001

GDS 15, 15 item Geriatric Depression Scale; CCDD, Center for Cognitive Disorders and Dementia. Missing outcome data in the post-intervention cohort: Brescia 23 patients; Rome 87 patients; Perugia 15 patients. *p-value referred to comparison between pre- and post-intervention. ^†^GDS 15 was introduced post-intervention.

Variations among HAs in process indicators are reported in Supplementary Table 3. The proportion of patients receiving diagnostic procedures increased post-intervention in all HAs in a different way: the proportion of patients who received two or more visits per year by the FP was lower in Milan (35%, 95% CI 32.0–38.3%) than in other HAs (ranging from 60 to 75%); the frequency of a cognitive test performed by FPs has grown less in Florence compared to Milan and Bologna (52 vs. 100%); prescription of a brain CT or MRI increased post-intervention in all HAs, however it remained lower in Milan (32.0%, 95% CI 28.9–35.2%) and in Florence (38.8%, 95% CI 33.2–44.7%) compared to Brescia (89.8%, 95% CI 85.1–93.4%). In Rome, FPs did not prescribe brain CT pre-intervention and prescribed it to 62% of patients post-intervention. Referral to a CCDD specialist increased in Brescia (97% of patients were referred), whereas it decreased in the other HAs (ranging from 62 to 87%).

Table 3 shows the results on the outcome indicators. The educational intervention likely resulted in a reduction in the time interval between first FP consultation and diagnosis compared with pre-intervention (mean difference −1 month, 95% CI −1.49 to −0.51) (*p* < 0.001). A diagnosis of early cognitive decline was confirmed more often post- than pre-intervention (41.7%, 95% CI 39.6–43.9% vs. 30.8%, 95% CI 28.6–33.0%) (*p* < 0.001). Fewer diagnoses of Alzheimer’s disease dementia and other dementias (frontotemporal dementia and Parkinsonism) were recorded in the post-intervention compared with pre-intervention (13.7%, 95% CI 12.2–15.3% vs. 16.6%, 95% CI 14.9–18.5%; and 4.5%, 95% CI 3.7–5.5% vs. 7.8%, 95% CI 6.6–9.2%). Compared with pre-intervention, more diagnoses of vascular dementia (12.6%, 95% CI 11.2–14.1% vs. 10.1%, 95% CI 8.7–11.6%), and mixed dementia (13.7%, 95% CI 12.2–15.2% vs. 9.0%, 95% CI 7.6–10.3%) were observed in the post-intervention. A lower number of unspecified dementia diagnoses were recorded in the post- compared with the pre-intervention (7.2%, 95% CI 6.2–8.4% vs. 18.5%, 95% CI 16.7–20.4%) (*p* < 0.001). The proportion of patients with a suspected cognitive decline that reverted to normal cognition during follow-up was around 6% both in the pre- and post-intervention. Missing outcome data was low in the post-intervention (around 6% of the included patients, as reported in Table 2’s caption). The proportion of patients who received a diagnosis directly by FPs was 24.7% in the pre-intervention, 39.8% in the post-intervention (data not shown).

**TABLE 3:** Time interval between first FP consultation and diagnosis of cognitive decline, and number (%, 95% CI) of diagnosis during follow-up, in the pre- and post-intervention.

	Pre-intervention N. 1,708	Post-intervention N. 2,028	*P*-value*
Time interval between first FP consultation and diagnosis months, mean (SD)	8.0 (8.0)	7.0 (7.0)	<0.001
IQR	2.6–13.4	2.3–11.7	
**Diagnosis**			
Early cognitive decline	526 (30.8, 28.6–33.0)	846 (41.7, 39.6–43.9)	<0.001
Alzheimer’s disease dementia	284 (16.6, 14.9–18.5)	278 (13.7, 12.2–15.3)	0.01
Vascular dementia	172 (10.1, 8.7–11.6)	256 (12.6, 11.2–14.1)	0.01
Mixed dementia	153 (9.0, 7.6–10.3)	278 (13.7, 12.2–15.2)	<0.001
Other dementias	134 (7.8, 6.6–9.2)	92 (4.5, 3.7–5.5)	<0.001
Unspecified dementia	316 (18.5, 16.7–20.4)	147 (7.2, 6.2–8.4)	<0.001
Reverted to normal cognition	104 (6.1, 5.1–7.3)	114 (5.6, 4.7–6.7)	0.54

FPs, family physicians; IQR, interquartile range. *p-value referred to comparison between pre- and post-intervention.

Variations in outcomes indicators among HAs are shown in Supplementary Table 4. Reduction in the time interval between first FP consultation and diagnosis was evident in all the HAs, except in Florence and Bologna, where the interval remained stable. FPs identified a high number of patients with early cognitive decline post-intervention in all the HAs, excluding Brescia and Milan where the number was almost stable. Observed changes in diagnosis distribution were different among the HAs, however the number of patients who received a diagnosis of unspecified dementia decreased substantially post-intervention in all HAs.

Interventions prescribed by FPs are listed in Table 4. The observed number of prescriptions of neuroleptics and antidepressants significantly decreased post-intervention, respectively from 18.0% (95% CI 16.2–19.9%) to 5.9% (95% CI 4.9–7.0%) (*p* < 0.001), and from 42.7% (95% CI 40.3–45.1%) to 35.1% (95% CI 33.0–37.2%) (*p* < 0.001), while prescriptions of benzodiazepines remained unchanged (21 and 22%, respectively). Compared with pre-intervention, more advice of physical rehabilitation were recorded post-intervention (5.4%, 95% CI 4.5–6.5% vs. 2.6%, 95% CI 1.9–3.5%) (*p* < 0.001), as for cognitive rehabilitation (11.3%, 95% CI 9.9 to 12.7 vs. 4.7%, 95% CI 3.7–5.7) (*p* < 0.001). There was also a substantial increase in prescriptions of day center from 2.9% (95% CI 2.1–3.8%) pre-intervention to 7.4% (95% CI 6.3–8.6%) post-intervention.

**TABLE 4:** Number (%, 95% CI) of patients with cognitive decline prescribed interventions, in the pre- and the post-intervention.

	Pre-intervention N. 1,708	Post-intervention N. 2,028	*P*-value*
Neuroleptics	307 (18.0, 16.2–19.9)	120 (5.9, 4.9–7.0)	<0.001
Benzodiazepines	360 (21.1, 19.2–23.0)	448 (22.1, 20.3–23.9)	0.45
Antidepressants	729 (42.7, 40.3–45.1)	712 (35.1, 33.0–37.2)	<0.001
Physical rehabilitation	45 (2.6, 1.9–3.5)	110 (5.4, 4.5–6.5)	<0.001
Cognitive rehabilitation	80 (4.7, 3.7–5.7)	229 (11.3, 9.9–12.7)	<0.01
Day center	49 (2.9, 2.1–3.8)	150 (7.4, 6.3–8.6)	<0.001

*p-value referred to comparison between pre- and post-intervention.

Variations in the prescription of interventions across HAs are shown in Supplementary Table 5. Post-intervention prescriptions of neuroleptics decreased in all HAs except in Brescia, antidepressants decreased in all HAs except in Brescia and Perugia where increased post-intervention, and prescriptions of benzodiazepines did not change, except in Brescia where increased and in Rome where decreased. There was variation in rehabilitation and day center participation among the HAs with high increase post-intervention in Brescia and Bologna. Rome had a high increase in day center participation and Perugia in cognitive rehabilitation post-intervention.

## Discussion

### Summary of main results and comparison with previous research in the field

Our results show an overall successful effect of an educational intervention addressed to FPs on detection and management of cognitive decline, as suggested by changes in selected process and outcome indicators. The number of visits per year for cognitive assessment by FPs increased, and the time from the first suspicion of cognitive decline to a confirmed diagnosis or its exclusion decreased post-intervention. FPs used cognitive and mood examination tests to evaluate patients with symptoms of cognitive decline or behavioral symptoms, confirming the usefulness and feasibility of these tests in primary care. The number of patients referred to a CCDD specialist was lower in the post- (81%) compared to pre-intervention (90%), suggesting that FPs became more confident in their evaluation using appropriately objective measures. We interpret this result as an improvement in referral behavior through a more careful selection of referred patients and a major awareness and expertise of FPs in recognizing early symptoms of cognitive decline, registering promptly clinical impressions or diagnoses in medical records in a standardized way and in managing relative disturbances after the educational intervention.

Moreover, even if the mean follow-up length was shorter in the post-intervention in all HAs, an increased number of suspected cases of cognitive decline was documented, compared to the pre-intervention and conversely, the number of unspecified dementias decreased significantly in all HAs after the intervention. In our opinion, a major identification of cases in early stage cognitive decline and a more precise definition of the diagnosis represent together results of an improved collaboration between FPs and specialists, promoted by the educational intervention, a successful strategy proposed by previous reports (3, 8, 24). In comparison with the study of Veneziani, conducted in 2011 on a sample of 131 Italian FPs from Southern Italy, where 53% of the sample declared not to use specific tests or protocols to diagnose or administer cognitive tests (8), in our study diagnostic procedures resulted largely overlooked in the pre-intervention, and increasingly applied after the intervention.

Regarding medication prescriptions, neuroleptics significantly decreased in post-intervention, whereas benzodiazepines remained stable and their prescription was relatively high (around 20%). Since in the pre-intervention a major number of participants was identified in a more advanced stage of the disease, a larger use of neuroleptics in the pre- compared to the post-intervention could also reflect participants’ characteristics. A lower neuroleptics prescription rate was appropriate and in line with local indications of each HA. Moreover, it is worth noting that since 2017 the Italian Medicines Agency (Agenzia Italiana del Farmaco, AIFA) strengthened the exclusion rules of FPs from the right of prescribing antipsychotics in dementia for side effects frequently reported in these patients. Conversely, despite the application of an educational session on inappropriateness and warning on benzodiazepine use in cognitive decline, these medicines remained largely prescribed in primary care. A high rate of benzodiazepine prescription has been already reported in Italy and in different countries, especially in nursing-home settings (12, 25, 26). These results highlight the need to reinforce recommendations and warnings regarding the use of benzodiazepines in primary care. The implementation of non-pharmacological interventions was underrepresented even after the educational intervention, thus with a limited overall impact on the clinical pathway of dementia. At least in part, the missing use of physical and cognitive rehabilitation and day center was due to the seemingly poor availability and accessibility of community-based dementia services, including long-term care.

### Differences among health authorities and interpretation

The educational intervention to FPs aimed to reduce variations in practice, and highlight differences that could stimulate interventions aimed to bring the worst situations to the best ones. The number of visits per year performed by FPs for cognitive decline increased in all HAs after the educational intervention, the change was not relevant in the HA of Perugia where the number of visits was already high in the pre-intervention period. The application of diagnostic procedures by FPs in case of suspected cognitive decline was most evident in centers where these procedures were previously neglected. In Rome and Perugia increased mainly the application of cognitive examination tests, in Rome increased consistently the neuroimaging prescriptions. Thus, overall results obtained in Rome may reflect the absence of a previous local DTCP at the study starting and the acquisition of competencies and confidence in diagnostic procedures by the FPs after the intervention.

Differently from other sites, the proportion of patients prescribed neuroleptics remained stable and was relatively high in Brescia (22%), as the prescription of benzodiazepines (40%). On the other side, the proportion of patients participating in rehabilitation and day center activities increased in Brescia post-intervention, highlighting a major use of non-pharmacological intervention in this HA. Overall, since DTCPs do not vary significantly in content, differences in results among HAs may depend mainly on pre-intervention habits in each local HA, and the availability or accessibility to specialized healthcare services and community-based support services.

Regional variability and some inequalities in terms of available resources and services for people with dementia were expected. Available data based on a national survey (27) showed discrepancies in the availability of dementia services among regions (e.g., in the number of day-care services that was superior from North to Southern Italy), indicating that access to health care may be restricted not only because of financial reasons, but also because of geographic barriers, waiting times and possibly other reasons. The variability depends at least in part on the organization of the Italian National Health System that is structured at national, regional, and local level. Regions have substantial autonomy in determining the macrostructure of their local health systems. Tracing available health and social-health services for people with dementia is part of the program of National dementia Plans (15).

### Strengths and limitations

The study had the advantage to involve a wide range of FPs, patients, and experts in the field of dementia in each HA belonging to different Italian regional communities, thus promoting a joint effort for the entire community to counteract dementia. The commitment in the study participation by each HA may have sensitized to update or disseminate their DTCP/guidelines as in Milan, Bologna, Florence and Rome, or to elaborate new regional plans as in Perugia. The educational intervention followed specific guidelines and the program was shared among experts and presented in a face-to-face meeting that permitted direct interaction with FPs, promoting their interest and awareness toward the problem, providing them with examples of practical application of standardized procedures, and facilitating their comprehension. There is previous evidence that educational interventions to FPs may improve professional practice also in this specific context (28–30), especially when baseline performance is low, the source is a supervisor, colleague, or an opinion leader, the content is presented more than once with short meetings, in both verbal and written formats providing additional take-home material, when the course is inclusive of both explicit targets and an action plan, and when a shorter follow-up is applied (31, 32).

A main limitation regards the assessment of the educational intervention effect performed only after about 1 year, leaving out a delayed verification of long-lasting learning effect. Moreover, the pre-post design of the study lacks a control group, limiting the strength of the evidence of a cause-effect relationship. Finally, the study was performed in a pre-COVID era, reflecting in part different health priorities and approaches compared to the current ones (33–35).

## Conclusion

The success of local dissemination and implementation of DTCP for cognitive decline is likely attributable to an interactive involvement of CCDD specialists and FPs in the educational sessions. Interventions to improve general practice management of patients with cognitive decline may influence referral behavior (number and quality of referrals), CCDD management of patients, and patient outcomes. Thus, further initiatives of DTCP dissemination and implementation should promote strategies to enhance interactions between primary and secondary care optimizing the collaboration between FPs and specialists.

## Data availability statement

The original contributions presented in the study are publicly available. This data can be found here: https://doi.org/10.5281/zenodo.7303817.

## Ethics statement

The studies involving human participants were reviewed and approved by Ethical Committee of the Carlo Besta Foundation and Neurological Institute. The patients/participants provided their written informed consent to participate in this study.

## Author contributions

GL and EC supervised the findings, conducted the literature search, wrote the original draft, and reviewed the final draft. DA performed data analysis and created the figure. AF, CMo, IP, and SP performed quality check of collected data and contributed to results interpretation. GBF, SS, DP, LR, and GS conceived and planned the experiment and reviewed the final draft. SL supervised the findings. CMu conceived and planned the experiment. FP conceived and planned the experiment, and supervised the findings. FT and PT conceived and planned the experiment and contributed to the interpretation of the results. LP contributed to the design and implementation of the research and reviewed the final draft. GF conceived and planned the experiment design, project administration, wrote the original draft, and reviewed and edited the final draft. All authors contributed to manuscript revision, read, and approved the submitted version.

## Abbreviations

FP: family physician
HA: Health Authority
CCDD: Center for Cognitive Disorders and Dementia
DTCP: Diagnostic and Therapeutic Care Pathway.
