# Supplementary material for: Educational interventions to improve detection and management of cognitive decline in primary care—An Italian multicenter pragmatic study
Source: Front Psychiatry. 2022 Nov 24;13:1050583. doi: 10.3389/fpsyt.2022.1050583 (PMC9731677; doi:10.3389/fpsyt.2022.1050583)
Supplement: Supplementary file 1 [file Data_Sheet_1.PDF]

frontiers

Supplementary Material

Supplementary Table 1. Local research team and resources available for each health authority at the study start

Referral CCDD	Local HA	Resources available and references
Carlo Besta Foundation and Neurological Institute	Azienda Sanitaria Locale Milano	Percorso Preventivo-Diagnostico-Terapeutico-Assistenziale Riabilitativo (PDTAR) per la popolazione e i pazienti con demenza<br>*https://fondazioneallinearesanitaesalute.org/wp-content/uploads/1-PDTAR-ASL-Milano-ATS-formazione-novembre-2016.pdf*
University of Brescia	Agenzia della Tutela della Salute Brescia	Percorso diagnostico-terapeutico-assistenziale decadimento cognitivo/demenza<br>*https://www.asst-spedalicivili.it/upload/spedalicivili_brescia/gestionedocumentale/PDTADecadimentocognitivo-demenza_784_17656.pdf?*
Institute of Neurological Sciences of Bologna	Agenzia sanitaria e sociale regionale, Regione Emilia-Romagna	Percorso Diagnostico-terapeutico-assistenziale nelle demenze<br>*http://bur.regione.emilia-romagna.it/bur/area-bollettini/bollettini-in-lavorazione/n-42-del-07-02-2019-parteseconda.2019-02-06.4576086107/recepimento-linee-di-indirizzonazionali-sui-percorsi-diagnostico-terapeutici-assistenziali-pdtaper-le-demenze-e-linee-di-indirizzo-nazionali-sulluso-dei-sistemiinformativi-per-caratterizzare-il-fenomeno-delle-demenze/all.2*
University of Florence	Azienda Sanitaria Locale Firenze Toscana Centro (Associazione GEA)	Linee Guida Consiglio Sanitario Regionale Sindrome Demenza: diagnosi e trattamento<br>*http://www.regione.toscana.it/documents/10180/320308/Sindrome+demenza.pdf/958f90f9-f94e-4095-a03a-d51293b9d47e?version=1.0*
IRCCS Santa Lucia Foundation	Azienda Sanitaria Locale Roma3	Determinazione Dirigenziale n. 657<br>*http://www.aslroma3.it/servizi-dalla-a-alla-z/a/alzheimer/*
University of Perugia	Azienda Sanitaria Locale Umbria 1	Piano regionale cronicità<br>*http://www.regione.umbria.it/documents/18/704709/DGR+902-17.pdf/730e2d61-0773-41cb-89b1-a82460c1a975*

CCDD: Center for Cognitive Disorders and Dementia; HA: health authority

Supplementary Table 2: Baseline characteristics of patients identified by FPs as having cognitive decline stratified by regional health authority and pre- post-educational intervention

	**Milan**		**Brescia**		**Bologna**		**Florence**		**Rome**		**Perugia**		**Total**	
	FPs No.64<br>Patients No. 1263		FPs No. 40<br>Patients No. 549		FPs No. 40<br>Patients No. 556		FPs No. 40<br>Patients No. 546		FPs No. 40<br>Patients No. 480		FPs No. 30<br>Patients No. 342		FPs No. 254<br>Patients No. 3736	
	PRE<br>N. 407	POST<br>N. 856	PRE<br>N. 323	POST<br>N. 226	PRE<br>N. 294	POST<br>N. 262	PRE<br>N. 255	POST<br>N. 291	PRE<br>N. 240	POST<br>N. 240	PRE<br>N. 189	POST<br>N. 153	PRE<br>N. 1708	POST<br>N. 2028
**Gender (N.,%)**<br>Men	125<br>(31)	318<br>(37)	126<br>(39)	110<br>(49)	120<br>(41)	101<br>(38)	82<br>(32)	107<br>(37)	86<br>(36)	99<br>(41)	76<br>(40)	56<br>(37)	615<br>(36)	791<br>(39)
Women	282<br>(69)	538<br>(63)	197<br>(61)	116<br>(51)	174<br>(59)	161<br>(62)	173<br>(68)	184<br>(63)	154<br>(64)	141<br>(59)	113<br>(60)	97<br>(63)	1093<br>(64)	1237<br>(61)
**Age**<br>Median<br>IQR	80<br>76 – 84	78<br>73 – 83	80<br>75 – 85	80<br>76 – 86	79<br>74– 84	79<br>74– 83	82<br>77 – 86	82<br>76 – 86	74<br>69 – 77	75<br>70 – 79	83<br>79 – 87	79<br>73 – 84	80<br>75– 85	79<br>73– 83
**CIRS total score**<br>Median<br>IQR	17<br>15-18	18<br>15-18	20<br>17-24	21<br>20-25	19(5)<br>17-22	22 (6)<br>19-25	19<br>17-20	19<br>17-20	18<br>16-19	18<br>16-19	18<br>17-21	21<br>17-21	18<br>16-21	18<br>17 - 22

CIRS: Cumulative Illness Rating Scale; FPs: family physicians; HA: Health Authority: IQR: interquartile range

Supplementary Table 3: Number (%) of patients prescribed diagnostic tests in primary care and referral to a CCDD specialist for cognitive assessment; all data stratified by regional health authority and pre- post-educational intervention.

	**Milan** N. 1263		**Brescia** N. 549		**Bologna** N. 556		**Florence** N. 546		**Rome** N. 480		**Perugia** N. 342		**Total** N. 3736	
	PRE N. 407	POST N. 856	PRE N. 323	POST N. 226	PRE N. 294	POST N. 262	PRE N. 255	POST N. 291	PRE N. 240	POST N. 240	PRE N. 189	POST N. 153	PRE N. 1708	POST N. 2028
≥ 2 visits per year for cognitive assessment	75 (18.4)	300 (35.0)	156 (48.3)	135 (59.7)	146 (49.6)	197 (75.2)	63 (24.7)	214 (73.5)	104 (43.3)	153 (63.7)	129 (68.2)	107 (69.9)	569 (33.3)	1106 (54.5)
At least one cognitive test	40 (9.8)	856 (100)	24 (7.4)	148 (65.5)	24 (8.2)	262 (100)	22 (8.6)	151 (51.9)	-†	224 (93.3)	1 (0.5)	142 (92.8)	111 (6.5)	1783 (87.9)
At least one GDS 15‡	-	812 (95.0)	-	39 (17.3)	-	167 (63.7)	-	137 (47.1)	-	20 (8.3)	-	47 (30.7)	-	1222 (60.3)
Blood tests	317 (77.9)	663 (77.5)	307 (95.0)	220 (97.3)	223 (75.8)	257 (98.1)	176 (69.0)	228 (78.3)	233 (97.1)	228 (95.0)	102 (54.0)	126 (82.3)	1358 (79.5)	1722 (84.9)
Brain CT or MRI	90 (22.1)	274 (32.0)	265 (82.0)	203 (89.8)	74 (25.2)	160 (61.1)	61 (23.9)	113 (38.8)	2 (0.8)	149 (62.1)	59 (31.2)	77 (50.3)	551 (32.3)	976 (48.1)
Referral to a CCDD specialist	404 (99.3)	748 (87.4)	291 (90.1)	219 (96.9)	223 (75.8)	186 (71.0)	212 (83.1)	224 (77.0)	238 (99.2)	148 (61.7)	166 (87.8)	124 (81.0)	1534 (89.8)	1649 (81.3)

CCDD: Center for Cognitive Disorders and Dementia; CT: Computed tomography; GDS 15: the 15 item Geriatric Depression Scale; FPs: family physicians; MRI: Magnetic Resonance Imaging

Note: Missing data in the post-intervention cohort: Brescia 23 patients; Rome 87 participants; Perugia 15 patients.

†In the healthcare authority of Rome, FPs had not used any cognitive examination test pre-intervention; ‡FPs had not used GDS 15 pre-intervention.

Supplementary Table 4: Time interval between the first FP consultation and diagnosis and number (%) of diagnosis during follow-up stratified by regional health authority and pre- post-educational intervention.

	Milan N. 1263		Brescia N. 549		Bologna N. 556		Florence N. 546		Rome N. 480		Perugia N. 342		Total N. 3736	
	PRE N. 407	POST N. 856	PRE N. 323	POST N. 226	PRE N. 294	POST N. 262	PRE N. 255	POST N. 291	PRE N. 240	POST N. 240	PRE N. 189	POST N. 153	PRE N. 1708	POST N. 2028
Time interval between first FP consultation and diagnosis, months mean (SD) range	11 (10) 0 –36	9 (4) 0 -18	7 (6) 0 – 28	4 (5) 0 – 18	5 (6) 0 – 30	7 (5) 0 – 16	7 (6) 0 – 30	8 (4) 1 – 17	9 (4) 1 – 18	8 (5) 1 – 18	8 (7) 0 – 39	6 (5) 1 – 19	8 (8) 0 – 39	7 (7) 0 – 19
**DIAGNOSIS AT FOLLOW-UP**														
Early cognitive decline	180 (44.2)	379 (44.3)	82 (25.4)	47 (20.8)	143 (48.6)	161 (61.4)	30 (11.8)	105 (36.1)	41 (17.1)	104 (43.3)	50 (26.5)	50 (32.7)	526 (30.8)	846 (41.7)
Alzheimer's disease dementia	69 (16.9)	137 (16.0)	42 (13.0)	31 (13.7)	6 (2.0)	10 (3.8)	53 (20.8)	30 (10.3)	91 (37.9)	51 (21.2)	23 (12.2)	19 (12.4)	284 (16.6)	278 (13.7)
Vascular dementia	52 (12.8)	112 (13.1)	33 (10.2)	36 (15.9)	32 (10.9)	18 (6.9)	22 (8.6)	29 (10.0)	20 (8.3)	38 (15.8)	13 (6.9)	23 (15.0)	172 (10.1)	256 (12.6)
Mixed dementia	37 (9.1)	98 (11.4)	27 (8.3)	59 (26.1)	13 (4.4)	15 (5.7)	27 (10.6)	47 (16.1)	39 (16.2)	19 (7.9)	10 (5.3)	40 (26.1)	153 (9.0)	278 (13.7)
Other dementias	28 (6.9)	50 (5.8)	13 (4.0)	14 (6.2)	4 (1.4)	0	51 (20.0)	19 (6.5)	0	2 (0.8)	38 (20.1)	7 (4.6)	134 (7.8)	92 (4.5)
Unspecified dementia	33 (8.1)	64 (7.5)	126 (39.0)	10 (4.4)	17 (5.8)	8 (3.0)	57 (22.3)	32 (11.0)	48 (20.0)	25 (10.4)	35 (18.5)	8 (5.2)	316 (18.5)	147 (7.2)

Supplementary Table 4 (Continued)

	**Milan** N. 1263		**Brescia** N. 549		**Bologna** N. 556		**Florence** N. 546		**Rome** N. 480		**Perugia** N. 342		**Total** N. 3736	
	PRE N. 407	POST N. 856	PRE N. 323	POST N. 226	PRE N. 294	POST N. 262	PRE N. 255	POST N. 291	PRE N. 240	POST N. 240	PRE N. 189	POST N. 153	PRE N. 1708	POST N. 2028
Reverted to normal cognition	8 (2.0)	16 (1.9)	0	18 (8.0)	79 (26.9)	50 (19.1)	15 (5.9)	23 (7.9)	1 (0.4)	1 (0.4)	1 (0.5)	6 (3.9)	104 (6.1)	114 (5.6)

FPs: family physicians; SD: standard deviation

Supplementary Table 5: Number (%) of patients with cognitive decline prescribed interventions in primary care stratified by regional health authority and pre- post-educational intervention.

	**Milan** N. 1263		**Brescia** N. 549		**Bologna** N. 556		**Florence** N. 546		**Rome** N. 480		**Perugia** N. 342		**Total** N. 3736	
	PRE No.407	POST No.856	PRE No.323	POST No.226	PRE No.294	POST No.262	PRE No.255	POST No.291	PRE No 240	POST No.240	PRE No.189	POST No.153	PRE No.1708	POST No.2028
Neuroleptics	59 (14.5)	20 (2.3)	65 (20.1)	50 (22.1)	23 (7.8)	16 (6.1)	71 (27.8)	9 (3.1)	51 (21.2)	17 (7.1)	38 (20.1)	8 (5.2)	307 (18.0)	120 (5.9)
Benzodiazepines	80 (20.0)	145 (16.9)	90 (27.9)	91 (40.3)	79 (26.9)	81 (30.9)	74 (29.0)	91 (31.3)	52 (21.7)	24 (10.0)	37 (19.6)	40 (26.1)	360 (21.1)	448 (22.1)
Antidepressants	112 (27.5)	110 (12.8)	145 (44.9)	122 (54.0)	123 (41.8)	116 (44.3)	158 (62.0)	171 (58.8)	125 (52.1)	114 (47.5)	66 (34.9)	79 (51.6)	729 (42.7)	712 (35.1)
Physical rehabilitation	8 (2.0)	17 (2.0)	3 (0.9)	33 (14.6)	3 (1.0)	14 (5.3)	15 (5.9)	31 (10.6)	14 (5.8)	10 (4.2)	2 (1.1)	5 (3.3)	45 (2.6)	110 (5.4)
Cognitive rehabilitation	53 (13.0)	111 (13.0)	3 (0.9)	47 (20.8)	8 (2.7)	32 (12.2)	2 (0.8)	5 (1.7)	13 (5.4)	20 (8.3)	1 (0.5)	14 (9.2)	80 (4.7)	229 (11.3)
Day Center	10 (2.5)	5 (0.6)	5 (1.5)	42 (18.6)	17 (5.8)	34 (13.0)	7 (2.7)	17 (5.8)	7 (2.9)	48 (20.0)	3 (1.6)	4 (2.6)	49 (2.9)	150 (7.4)
